# Electron, phonon and thermoelectric properties of Cu_7_PS_6_ crystal calculated at DFT level

**DOI:** 10.1038/s41598-021-98515-6

**Published:** 2021-09-24

**Authors:** B. Andriyevsky, I. E. Barchiy, I. P. Studenyak, A. I. Kashuba, M. Piasecki

**Affiliations:** 1 Faculty of Electronics and Computer Sciences, Koszalin University of Technology, Śniadeckich str. 2, 75453 Koszalin, Poland; 2 Inorganic Chemistry Department, Uzhhorod National University, Pidhirna str. 46, Uzhhorod, 88000 Ukraine; 3 Department of General Physics, Lviv Polytechnic National University, Bandera str. 12, Lviv, 79013 Ukraine; 4 Institute of Physics, Jan Dlugosz University of Częstochowa, Armii Krajowej str. 13/15, Czestochowa, Poland

**Keywords:** Energy science and technology, Materials science, Physics

## Abstract

The promising class of the environment-friendly thermoelectrics is the copper-based argyrodite-type ion-conducting crystals exhibiting just extraordinary low thermal conductivity below the glass limit associated with the molten copper sublattice leading to a softening of phonon modes. To explain why the argyrodite structure containing copper ions favors the low thermal conductivity, we have utilized the ab initio calculations of the electron, phonon, and thermoelectric properties of Cu_7_PS_6_ crystal in the framework of the density functional and Boltzmann transport theories. To obtain the reliable thermoelectric properties of Cu_7_PS_6_, we take into account the dependence of the electron effective mass m^*^ on the redundant carrier concentration *n*. We propose to use the Burstein–Moss effect for the calculation of the electron effective mass m^*^ of a semiconductor. We have found the strong nonlinear character of copper atom vibrations in Cu_7_PS_6_ which exceeds substantially the similar values for phosphorous and sulfur atoms. The large vibration nonlinearity of the copper atoms found in Cu_7_PS_6_ explains the diffusion-like heat transfer and the relatively low coefficient of the lattice thermal conductivity (κ = 0.7 W/(m K)), which is favorable to achieve the large thermoelectric figure of merit.

## Introduction

**Figure 1:**
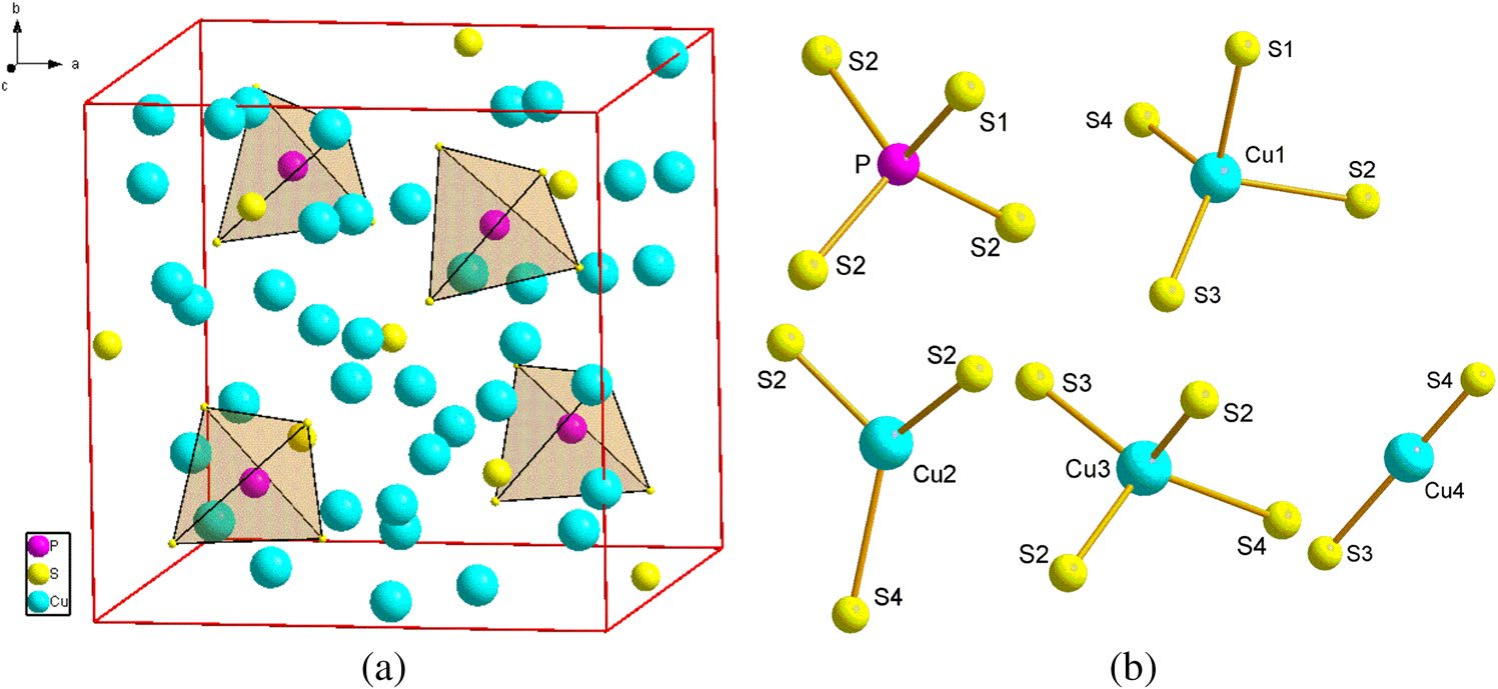
(**a**) The cubic space group *P*2_1_3 framework of Cu_7_PS_6_, (**b**) nearest coordination environment of P and Cu atoms.

Cu_7_PS_6_ compound belongs to the argyrodite-type solid electrolytes^1^. At low temperatures the α-modification of the crystal belongs to the orthorhombic space groups *Pmn*2_1_ (no. 31) (*T* ≤ 213 K) and *Pna*2_1_ (no. 33) (*T* ≤ 173 K)^2^. At room temperature, the crystal structure of Cu_7_PS_6_ belongs to the cubic space group *P*2_1_3 (No. 198) with lattice parameter *a* = 0.96706 nm and four formula units per unit cell (*Z* = 4), which is identical to the structure of β-Cu_7_PSe_6_^3^ (Fig. 1). The known solid electrolyte properties of Cu_7_PSe_6_ are due to the huge structural disorder of copper atoms/ions^4^. It was found that the high room temperature total conductivity of Cu_7_PSe_6_, σ = 0.4 S/cm, is about 90% due to the electronic component of conductivity^4,5^. The electric conductivity of Cu_7_PS_6_ in the temperature range 296–351 K was measured to be in the range 2 × 10^–5^–5 × 10^–5^ S/cm^3^ which is much smaller than that in Cu_7_PSe_6_^6^. Due to the same crystal structure of Cu_7_PSe_6_ and Cu_7_PS_6_ mentioned above the high structural disorder of copper atoms is expected in Cu_7_PS_6_, similarly like in Cu_7_PSe_6_^4^.

Recently, the effect of isovalent S^2−^ substitution for Se^2−^ in Cu_7_PSe_6_ on the crystal structure of the solid solution Cu_7_P(Se_1−x_S_x_)_6_ has been studied^7^. It was confirmed that the crystal structure of β-Cu_7_PSe_6_ transforms to the face-centered high-temperature γ-modification ($F\overline{4} 3m$) above 320 K and the promising thermoelectric and ion-conducting properties are observed only in this latter modification, where the cations are mobile, so the coper ion diffusion takes place. A similar transition for the homologous Cu_7_PS_6_ occurs at 510 K. One of the main results of this study is the anion ordering due to site preference of the sulfide ions. This leads to a stabilization of the high-temperature structure of Cu_7_P(Se_1−x_S_x_)_6_ even at lower temperatures. Thus, the isovalent substitution Se^2−^ by S^2−^ in Cu_7_P(Se_1−x_S_x_)_6_ allows the stabilization of the polymorph (γ-modification) with the most promising properties. This conclusion agrees with the results of the previous study of the conductivity of solid solution Cu_7_P(Se_1−x_S_x_)_6_, where the phase transition from the primitive cubic structure *P*2_1_3 to the face-centered one $F\overline{4} 3m$ was detected already in room temperature for the sulfur contents *x* ≥ 0.08^6^.

Thus, the face-centered symmetry $F\overline{4} 3m$, realized in Cu_7_PS_6_ and Cu_7_PSe_6_ crystals and their solid solution, is associated with the temperature-dependent copper ion diffusion, which introduces the structural disorder or leads to amorphization. This amorphization causes the coefficient of thermal conductivity lowering and as a consequence increases the thermoelectric figure of merit. On the other hand, the copper ion diffusion in the crystal creates in fact the copper vacancies, which lead to the appearance of the electron donor states in the band structure. In turn, these electron states may increase electric conductivity, which also improves the material's thermoelectric characteristics. Thus, one may expect the increased ion- and electron-conducting properties among the representatives of Cu_7_P(Se_1−x_S_x_)_6_, which may induce advanced thermoelectric and solid electrolyte properties. That is why the theoretical study of the electron and phonon properties of Cu_7_PS_6_ and Cu_7_PSe_6_ crystals and their solid solutions Cu_7_P(Se_1−x_S_x_)_6_ is a promising task, solving of which may deliver more information on how to improve the solid electrolyte and thermoelectric properties of materials by appropriate selection of the chemical composition.

The most important electronic characteristics of a crystal for their thermoelectric and photovoltaic applications are the bandgap *E*_g_, the effective electron mass m^*^, the carrier relaxation time τ, Seebeck coefficient α, and the coefficients of electric (σ) and thermal (κ) conductivities^8^. The ability to determine these characteristics for certain material compositions by using the theoretical methods is promising for the prediction of the main thermoelectric and photovoltaic characteristics (without having them synthesize) and evaluation of their possible practical applications. In the present study, we develop consistent and complex methods to determine the mentioned above material constants by calculations within the density functional and Boltzmann transport theories. To illustrate the effectiveness of our approach, the proposed methods have been employed for Cu_7_PS_6_ crystal and the obtained results have been compared with experimental data. We intentionally have chosen this crystal out of other contents of Cu_7_P(Se_1−x_S_x_)_6_ solid solutions, because, Cu_7_PS_6_ possesses the relatively large energy gap, *E*_g_ ≈ 2 eV, among other representatives of the group Cu_7_P(Se_1−x_S_x_)_6_, that is suitable for the study of the influence the extrinsic carriers on the electronic and thermoelectric properties of Cu_7_P(Se_1−x_S_x_)_6_ solid solutions. Further, having good consistency of our calculations with the available experimental data for Cu_7_PS_6_, in the next stage of our work, we intend to extend our calculations to a wide group of the argyrodite-type solid solutions to determine the chemical composition-structure-properties relations useful for finding the most effective thermoelectric material among this group.

## Results and discussion

### Electron band structure and related thermoelectric properties of Cu_7_PS_6_

**Figure 2:**
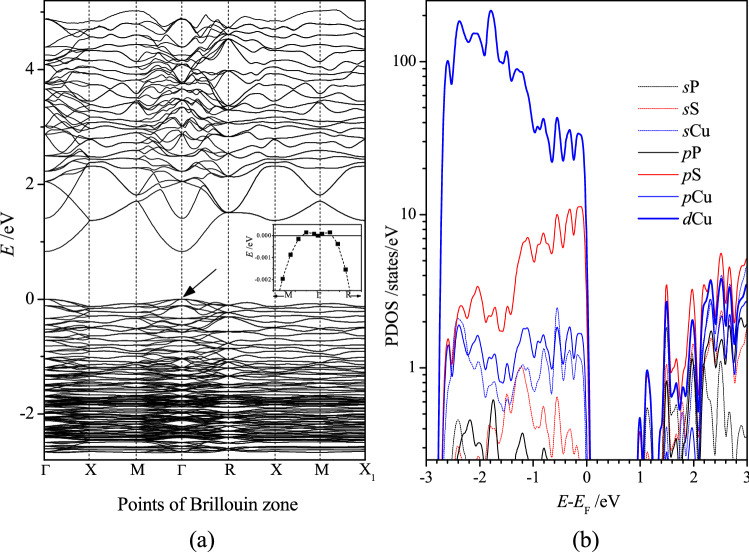
Band structure (**a**) and partial density of states (**b**) of Cu_7_PS_6_ for the symmetry space group no. 198 at the points Γ—000, X—010, M—½½0, R—½½½, X1—100 of BZ. The highest energy of the top valence band is placed at *E* = 0 eV. In the inset of (**a**), the enlarge *E*(*k*) dependence of the top valence band no. 236 (v236) in the vicinity of Γ-point (shown by arrow).

The value of the energy gap *E*_g_ = 0.83 eV obtained using the ordinary DFT approach (Fig. 2a) is more than twice smaller than the experimental one, *E*_g_ = 2.02 eV^9,10^. The thermoelectric properties of Cu_7_PS_6_ calculated using the energy gaps *E*_g_ = 0.83 eV and *E*_g_ = 2.02 eV have been found to be almost the same for different temperatures in the range *T* < 500 K, and therefore may be related to the extrinsic electrons in Cu_7_PS_6_. The mentioned above underestimation of the calculated energy gap *E*_g_ may be corrected using for example the DFT + U approach^11,12^, Heyd–Scuseria–Ernzerhof hybrid functional (HSE06)^13^, or modified Becke–Johnson exchange potential with L(S)DA correlation^14,15^. We used the HSE06 functional and the modified Becke–Johnson exchange potential (mBJ) and have obtained the calculated energy gap *E*_g_ close to the experimental one, *E*_g_ = 2.02 eV^9,10^.

The thermoelectric properties of the heavy degenerated wide energy gap semiconductors are determined mainly by the extrinsic charge carrier concentration in the conduction (*n*-type carriers) or valence (*p*-type carriers) bands and the energy gap value *E*_g_ = 2.0 eV of Cu_7_PS_6_, should not influence substantially these properties at the temperatures not much higher than the ambient one. Taking the later remark into account, we present the thermoelectric properties of Cu_7_PS_6_ calculated by the BoltzTrap2 code^16,17^ using the results of the ordinary DFT band structure calculations performed by VASP code^18–23^ with opt-B86b exchange-and-correlation functional^24^ and PAW-PBE pseudopotentials^23^ and applying the scissor factor *s* = 2.0 corresponding to the energy gap *E*_g_ = 2.0 eV^9,10^.

The highest valence bands of the crystal Cu_7_PS_6_ in the range 0 to − 3.0 eV are relatively flat (Fig. 2a). The highest valence band dispersion is observed at the points Γ and R of the Brillouin zone (Fig. 2a). The bottom conduction bands, located in the energy range of 0.83–5 eV, are characterized by the relatively large electron wave vector dispersion of energy *E*(*k*) in comparison to the top valence bands. This means that the electron effective masses m^*^ = *ħ*^2^/(d^2^*E*/d*k*^2^), as one of the main characteristics of semiconductors, for the bottom conduction bands are substantially smaller than the similar values for the top valence bands.

In view of the electric and thermoelectric properties of a material, the most significant energy ranges of the corresponding band structure are those neighboring to the energy gap *E*_g_. The top valence and bottom conduction bands of the crystal in the range – 3 to 3 eV are formed mainly by the *d*-electrons of copper and *p*-electrons of sulfur (Fig. 2b). This mainly is caused by the highest relative content of copper and sulfur atoms in Cu_7_PS_6_. The smallest PDOS of phosphorous is explained by the smaller it’s content in the formula unit Cu_7_PS_6_. The band structure is characterized by the relatively high hybridization of electronic states in the energy ranges close to the energy gap *E*_g_, which manifests itself in the similar PDOS maxima in the range of the top valence and bottom conduction bands (Fig. 2b). Thus the bonding electrons of the most numerous atoms copper and sulfur form mainly the electronic states, which may be relevant to the electron conductivity of Cu_7_PS_6_.

The effective masses of electrons m^*^_e_ and holes m^*^_h_ are essential parameters characterizing the mobility of electric charges in semiconductors and substantially influence the thermoelectric and photoelectric properties^25^. The effective masses of electrons and holes in Cu_7_PS_6_ have been calculated by utilizing the Effective Mass Calculator^26^ and by using the Burstein–Moss effect. In the latter case, the excess charge carriers (electrons or holes), associated with doping of semiconductors, cause the increase of energy gap^27,28^. The energy gap increase Δ*E*_g_, caused by the excess electrons, is equal to the Fermi energy change Δ*E*_F_ = Δ*E*_g_ depending on the carrier concentration *n* and the effective electron mass *m*^⁎^ in the conduction band,

1 $$\Delta E_{{\text{F}}} = \frac{{h^{2} }}{{8\pi^{2} m^{*} }}\left( {3\pi^{2} n} \right)^{2/3} ,$$

where *h* is Planck's constant. The energy gap increase Δ*E*_g_ may be caused by the excess or lack of electrons in the crystal unit cell. On the basis of the measured or calculated value Δ*E*_g_ = Δ*E*_F_ and using the relation (1) one can calculate the effective electron or hole masses, m^*^_e_ or m^*^_h_, corresponding to the ranges of conduction (c) or valence (v) bands. In the present study, the energy gap increase Δ*E*_g_ = Δ*E*_F_, caused by the excess electron concentration *n*, was simulated computationally and taken as the Fermi energy shift Δ*E*_F_ = *E*_F_ − *E*_cbm_, where *E*_F_ is the Fermi energy of the *n*-type semiconductors studied and *E*_cbm_ is the energy of the conduction band corresponding to the bottom conduction one of the nominal crystal. Similarly, in the case of the hole conductivity, the value of Δ*E*_F_ was taken as the Fermi energy shift Δ*E*_F_ = *E*_F_ − *E*_vbm_, where *E*_vbm_ is the energy of the valence band corresponding to the top valence one of the nominal semiconductor. The corresponding calculations have been performed for the excess electron and hole concentrations *n*, which corresponds to the *n*- or *p*-type semiconductors.

**Figure 3:**
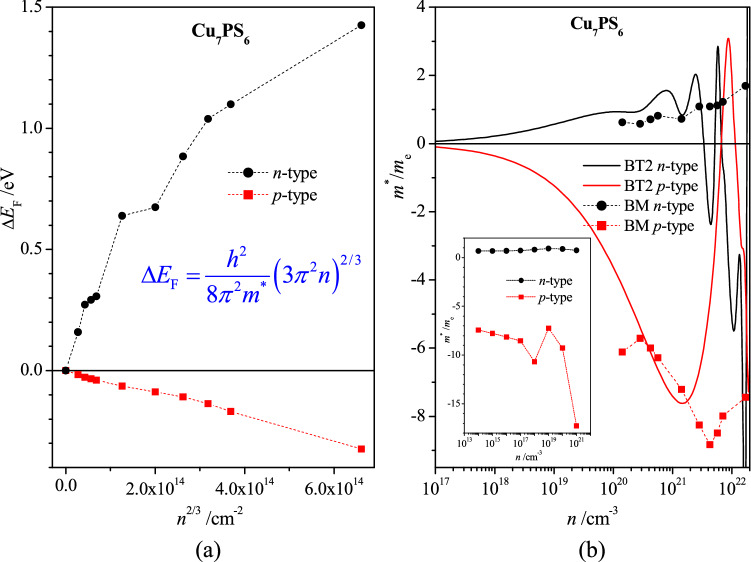
(**a**) Fermi level shift Δ*E*_F_ as a function of the excess electron (*n*-type) and hole (*p*-type) concentration *n*^2/3^ for Cu_7_PS_6_. (**b**) Effective electron mass m^*^/*m*_e_ as a function of the excess electron (*n*-type) and hole (*p*-type) concentration *n* calculated using the relation (1) and the data of Fig. 3a (points, BM), and from BoltzTraP2 Seebeck coefficient α using the relation (2) (solid lines, BT2). Signs of the effective masses m^*^ are chosen to be compatible with the effective mass definition m^*^ = *ħ*^2^/(d^2^*E*/d*k*^2^) for the band dispersions *E*(*k*) at Γ-point (Fig. 2a). In the inset, the effective mass m^*^, obtained on the basis of the relation (3), where the values μ and τ were calculated using AMSET code^30^ at the temperature 300 K (https://hackingmaterials.lbl.gov/amset/).

The calculated dependences of the Fermi level shift Δ*E*_F_ on the excess carrier concentration *n*^2/3^ reveal a quasi-monotonous behavior for the *n*- and *p*-type carriers in Cu_7_PS_6_, which is close to the linear one only for the *p*-type carriers (Fig. 3a). The corresponding effective masses *m*_e_^*^ and *m*_h_^*^ calculated by the relationship (1) as functions of the carrier concentration *n* values are presented by points in Fig. 3b. The mentioned quasi-linear dependence Δ*E*_F_(*n*^2/3^) for holes (Fig. 3a) result in the hole effective mass *m*_h_^*^ in the range of 6–9 *m*_e_ (Fig. 3b), where *m*_e_ is the free electron mass. The electron effective mass *m*_e_^*^, calculated in the same range of excess carrier concentration 1.4 × 10^20^–1.7 × 10^22^ cm^−3^, is placed in the range of 0.58–1.69 *m*_e_ (Fig. 3b). The value *m*_e_^*^ = 0.58 *m*_e_ is very close to that obtained for the first conduction band no. 237 at Γ-point of BZ by using Electron Mass Calculator, *m*_e_^*^ = 0.59 *m*_e_ (Supplementary Table 1). For both, electron and hole excess carriers, the averaged increase of the absolute value of the effective carrier mass |*m*_e_^*^| and |*m*_h_^*^| takes place in the range 1.4 × 10^20^–1.7 × 10^22^ cm^−3^ of the excess carrier concentration. This result is generally in agreement with the view of the conduction bands of Cu_7_PS_6_, where the decrease of the band dispersion d^2^*E*/d*k*^2^ is clear visible at an increase of energy (Fig. 2a), which corresponds to the increase of the electron effective mass m^*^ (there is no such clear change of the band dispersion d^2^*E*/d*k*^2^ in the range of the top group of valence bands (Fig. 2a).

The analysis of the electron/hole effective mass of Cu_7_PS_6_ presented above indicates that the effective mass of the conduction electrons is much smaller than the similar value for the holes. Thus, one may expect the corresponding differences in the physical values of Cu_7_PS_6_ depending on the effective electron mass.

Several thermoelectric properties of Cu_7_PS_6_ were calculated in the framework of DFT using the VASP and BoltzTraP2 codes. One of the main thermoelectric parameters is the Seebeck coefficient (α), which is proportional to the charge carrier effective mass m^*^, temperature *T*, and the inverse charge carrier density *n*^−2/3^^29^,

2 $$\alpha = \frac{{2k_{B}^{2} }}{{3e\hbar^{2} }}m^{*} T\left( {\frac{\pi }{3n}} \right)^{\frac{2}{3}} .$$

Here, *k*_B_ is Boltzmann’s constant, *e* is the electron charge, *h* is Planck’s constant. Thus, a large Seebeck coefficient is expected in material possessing large effective mass and small carrier concentration. Having calculated the Seebeck coefficient of Cu_7_PS_6_ by applying the VASP and BoltzTraP2 codes (Fig. 3), one has the possibility to calculate the effective mass m^*^ from the relation (2) (solid lines in Fig. 3b). Thus calculated effective mass m^*^ is comparable with that obtained by the method utilizing the Burstein–Moss effect in the limited ranges of the carrier concentration, 1 × 10^19^–3 × 10^21^ cm^−3^ for electrons and 1 × 10^20^–3 × 10^21^ cm^−3^ for holes (Fig. 3b). For smaller concentrations (*n* < 1 × 10^19^ cm^−3^ for electrons and *n* < 1 × 10^20^ cm^−3^ for holes), the decrease to zero of the absolute value of the carrier effective mass |m^*^| is observed (solid lines in Fig. 3b). This result is not reasonable because it does not agree with the limited electron and hole effective masses, |*m*_e_^*^| ≈ 0.6 *m*_e_ and |*m*_h_^*^| ≈ 6 *m*_e_, expected on the basis of the bottom conduction and top valence band energy dispersions d^2^*E*/d*k*^2^ of Cu_7_PS_6_ at Γ-point of BZ (Fig. 2a). That is why the effective mass m^*^, calculated using the Burstein–Moss effect, may be utilized for the proper calculations of the carrier mobility μ, relaxation time τ, and electric conductivity σ. Afterward, the power factor *PF* and the figure of merit *ZT*, as the main thermoelectric values, may be calculated using the BoltzTrap2 code. In the present study, the thermoelectric properties of Cu_7_PS_6_ were calculated using the new AMSET code^30^, which uses also the BoltzTrap2 one.

**Figure 4:**
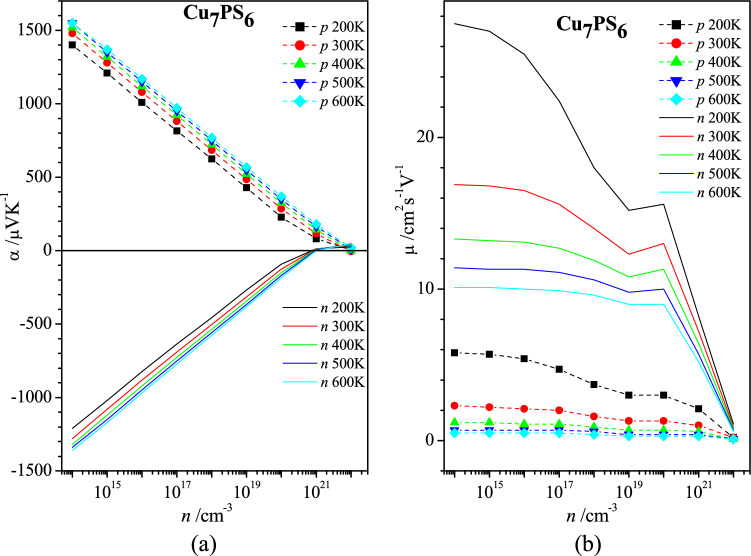
Dependences of (**a**) Seebeck coefficient α and (**b**) mobility μ on carrier concentration *n* for Cu_7_PS_6_ at the temperatures 200 K, 300 K, 400 K, 500 K, and 600 K for electron (*n*) and hole (*p*) types carriers.

According to the relation of the effective masses |*m*_h_^*^| >|*m*_e_^*^| mentioned above (Fig. 3b), the absolute value of the Seebeck coefficient α is larger for the *p*-type carriers of Cu_7_PS_6_, in comparison to the *n*-type one (Fig. 4a). The decrease of the Seebeck coefficient |α| is observed when the carrier concentration *n* increases. For every carrier concentration *n* the absolute value of Seebeck coefficient |α| increases with the increase in temperature (Fig. 4a). These features are in agreement with the relation (2).

We have found that the value of scattering rate *SR* ([*SR*] = s^−1^) of electric carriers (electrons and holes) in Cu_7_PS_6_ is determined mainly by the polar optical phonon scattering. The mechanism of the ionized impurity scattering prevails only for the heavy doped Cu_7_PS_6_ (*n* ≥ 10^21^ cm^−3^). The scattering rate related to the acoustic deformation potential is found to be two orders of magnitude smaller than that related to the polar optical phonon mechanism. Thus the carrier scattering mechanisms associated with the acoustic deformation potential, polar optical phonons, and ionized impurities were taken into account at the properties calculations using AMSET code. One of these calculated properties is the carrier mobility μ, calculated for the different electron (*n*) and hole (*p*) carrier concentrations *n* and temperatures (Fig. 4b). As was expected for the extrinsic semiconductor, the carrier mobility decreases with increasing the carrier concentration and temperature.

Having calculated by the AMSET code the mobility μ, and the scattering rate, *SR* = τ^−1^, one can obtain the effective mass m^*^ on the basis of the known relation,

3 $$\tau = \frac{{m^{*} }}{e}\mu ,$$

where *e* is the electron charge. Such calculated effective mass m^*^ as a function of the carrier concentration *n*, corresponding to the temperature 300 K, is presented in the inset of Fig. 3b. This dependence m^*^(*n*) is in satisfactory agreement with that obtained by using the Burstein–Moss effect (Fig. 3b). Thus, in Cu_7_PS_6_, the hole effective mass *m*_h_^*^ is about one order of magnitude larger than the electron effective mass *m*_e_^*^.

**Figure 5:**
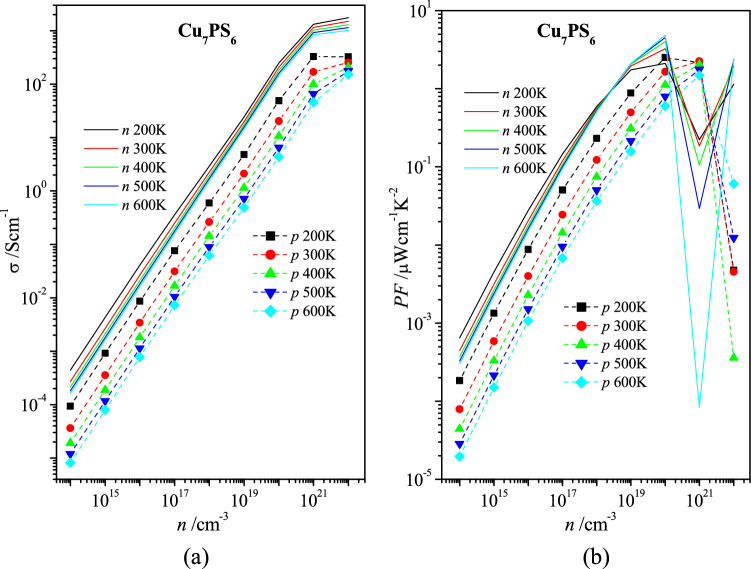
Dependences of (**a**) electric conductivity σ and (**b**) power factor *PF* on carrier concentration *n* for Cu_7_PS_6_ at the temperatures 200 K, 300 K, 400 K, 500 K, and 600 K for electron (*n*) and hole (*p*) types carriers.

The monotonous increase of specific conductivity σ with an increase of carrier concentration *n* is expected in the whole range of the concentration *n* for both types of charge carriers (Fig. 5a). Here, the *n*-type conductivity of Cu_7_PS_6_ is at least one order of magnitude larger than the *p*-type one. A similar monotonous increase takes place for the concentration dependences of the power factor *PF* (*PF* = α^2^σ) up to *n* ≈ 10^20^ cm^−3^ (Fig. 5b). The clear extremum-like concentration dependences in the range 10^20^–10^22^ cm^−3^ of the power factor *PF* for different temperatures take place for the *p*-type carriers (Fig. 5b). The large drop of the power factor for the *n*-type carriers at *n* = 10^21^ cm^−3^ and the extremum-like concentration dependences *PF*(*n*) are caused by the very small Seebeck coefficient α in the range 10^20^–10^22^ cm^−3^ (Fig. 4a). Also, the opposite characters of the temperature dependences *PF*(*T*) for the *n*-type carries are observed in the concentration range *n* < 3 × 10^18^ cm^−3^ and *n* > 3 × 10^18^ cm^−3^ (Fig. 5b).

For metals and extrinsic semiconductors, the temperature dependence of the specific resistivity ρ(*T*) (ρ = σ^−1^) may be fitted by the relationship,

4 $$\rho = \rho_{0} \left( {\frac{T}{{T_{0} }}} \right)^{p} ,$$

where ρ_0_ is the resistivity at the temperature *T*_0_ and *p* is the power index depending on the features of the carrier scattering^31^. The power indices *p* calculated for two carrier concentrations *n* (10^14^ and 10^21^ cm^−3^) in two temperature regions (200–240 K and 540–600 K) for the *n*- and *p*-type Cu_7_PS_6_ are presented in Table 1. Here worth reminding that the power index *p* = 1 corresponds to the linear temperature dependence of resistivity in the ideal pure metal, when the power index *p* = 0 means no temperature dependence of resistivity. More strong temperature dependence of the specific resistivity ρ(*T*) of Cu_7_PS_6_ is observed for the *p*-type conductivity in comparison with the *n*-type one (Table 1).

**Table 1:** Power index *p* of the temperature dependence of specific resistivity ρ(*T*) (4) for the extrinsic *n*- and *p*-type electric carriers in Cu_7_PS_6_ calculated by using AMSET code, v0.4.11^30^ (https://hackingmaterials.lbl.gov/amset/).

*n*/cm^−3^	*n*-type				*p*-type			
	10^14^		10^21^		10^14^		10^21^	
*T*/K	200–240	540–600	200–240	540–600	200–240	540–600	200–240	540–600
*p*	1.28	0.63	0.30	0.56	2.37	2.03	1.63	1.98

**Figure 6:**
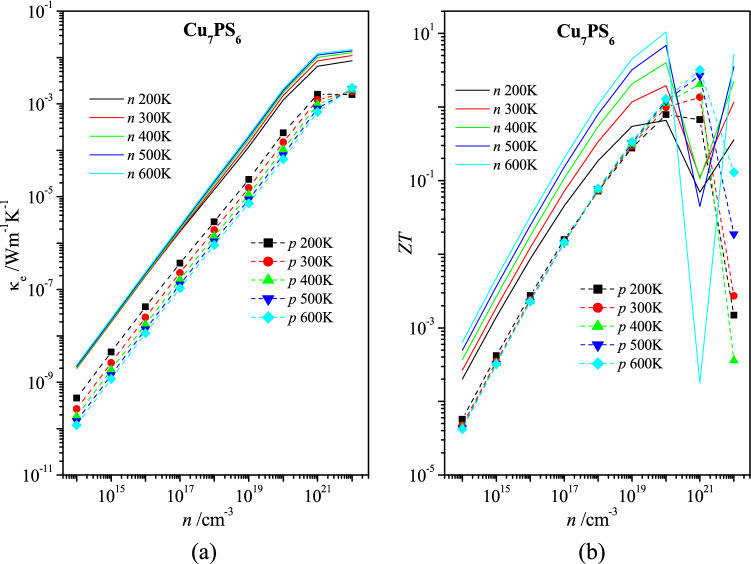
Dependences of (**a**) coefficient of electron thermal conductivity κ_e_ and (**b**) figure of merit *ZT* on carrier concentration *n* for Cu_7_PS_6_ at the temperatures 200 K, 300 K, 400 K, 500 K, and 600 K for electron (*n*) and hole (*p*) types carriers.

To obtain the thermoelectric figure of merit *ZT* of material,

5 $$ZT = \frac{{\alpha^{2} \sigma T}}{\kappa },$$

one has to know the coefficient of thermal conductivity κ. The later value is a sum of the corresponding electron and lattice (phonon) components, κ = κ_e_ + κ_ph_. The coefficient of electron thermal conductivity κ_e_ of Cu_7_PS_6_ is determined on the basis of the corresponding specific electric conductivity σ using the Wiedemann–Franz Law,

6 $$\kappa_{{\text{e}}} = L{\sigma}T,$$

where *L* is the Lorenz number, equal to 2.45﻿ × 10^–8^ W Ohm K^−2^, *T* is temperature. The calculated thermal conductivity κ_e_ does not exceed the values κ_e_ = 2 × 10^–3^ Wm^−1^ K^−1^ for electron carriers and κ_e_ = 2 × 10^–4^ Wm^−1^ K^−1^ for holes even for the relatively high carrier concentration *n* = 10^20^ cm^−3^ (Fig. 6a).

To obtain the thermoelectric figure of merit *ZT* of Cu_7_PS_6_ one has to have the total coefficient of thermal conductivity κ = κ_e_ + κ_ph_. Thus, estimation of the coefficient of lattice thermal conductivity κ_ph_ is necessary to obtain the reliable value of the total coefficient of thermal conductivity κ and finally the figure of merit *ZT*.

### Lattice-based thermal conductivity of Cu_7_PS_6_

Two models of thermal conductivity in solids have been used to estimate the coefficient of the lattice thermal conductivity of Cu_7_PS_6_. The first one is phonon-based and the second one is diffusion-based^32^. The phonon-based model is implemented in the Phono3py code^33^ and is usually applied to the crystalline solids of the high and moderate coefficient of thermal conductivity. In turn, the diffusion-based model of Allen and Feldman^34–36^ is applied more successfully to the amorphous or polycrystalline materials possessing a relatively small coefficient of thermal conductivity^32^. The Cu_7_PS_6_ crystal is close to the latter type of materials due to the weak bonding of copper atoms in the structure, similar to that found in Cu_7_PSe_6_^4^.

In the phonon based model, the coefficient of thermal conductivity is calculated by the principle relation,

7 $$\kappa_{ph}^{(1)} = \frac{1}{3}cv_{g}^{2} \tau ,$$

where *c* is the specific heat, *v*_g_ is the phonon group velocity, and τ is the phonon relaxation time. In the diffusion-based model, the maximum thermal conductivity is calculated by the relation,

8 $$\kappa_{ph}^{(2)} = \frac{{n^{\frac{1}{3}} k_{{\text{B}}} }}{\pi }\omega_{{{\text{avg}}}} ,$$

where *n* is the density of atoms, ω_avg_ is the averaged oscillator frequency, *k*_B_ is Boltzmann constant^32^. In the present study, the averaged oscillator frequency ω_avg_ of Cu_7_PS_6_ has been obtained from the calculated vibration density of states obtained by using the lattice and molecular dynamics (Supplementary Figs. 1, 2).

**Figure 7:**
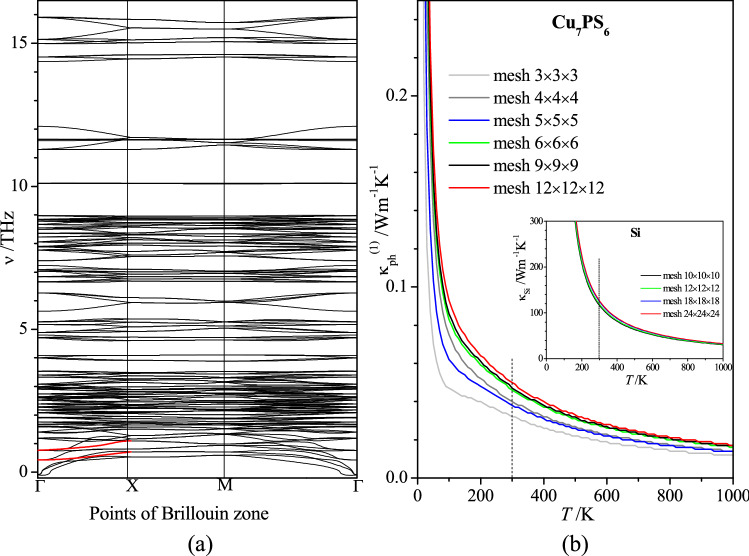
(**a**) Dispersion of the phonon modes ν(*q*) and (**b**) temperature dependences of lattice thermal conductivity coefficient κ_ph_^(1)^ of Cu_7_PS_6_ obtained using different *q*-meshes of the inverse lattice. In the inset, temperature dependences of lattice thermal conductivity coefficient κ_ph_^(1)^ for different *q*-meshes of silicon crystal obtained using the same Phono3py code, v.2.0.0, https://phonopy.github.io/phono3py/index.html.

The dispersion of phonon bands was calculated using the VASP and Phonopy^37^ codes and the results are presented in Fig. 7a. From the phonon dispersion, we notice that there exist soft phonon optical modes with frequency decreasing near the BZ center, as highlighted in red in Fig. 7a. A similar feature of the phonon dispersion was observed in Ref.^38^. It was commented that such optical phonon softening induces strong phonon anharmonicity^39,40^. The optical phonon softening detected (Fig. 7a) is in agreement with the anharmonicity of the copper atoms in Cu_7_PS_6_ observed in the molecular dynamics (Supplementary Fig. 1b). Small negative frequencies in the vicinity of Γ-point (Fig. 7a) show the structural instability of Cu_7_PS_6_, which may be associated with the increased mobility of the copper atoms. This feature implies the low lattice thermal conductivity of Cu_7_PS_6_. It should be mentioned that the presence of these negative frequencies is independent of the atoms displacements amplitude *A* in the range 0.015–0.1 Å, the phonon *q*-mesh density (12 × 12 × 12 and 24 × 24 × 24), or the electron *k*-mesh density (2 × 2 × 2, 4 × 4 × 4, and 6 × 6 × 6).

The coefficient of lattice thermal conductivity κ_ph_^(1)^ of Cu_7_PS_6_ was calculated using VASP and Phono3py^33^ codes (Fig. 7b). In accordance with the expectation, we have obtained the extremely small value of the coefficient of the lattice thermal conductivity of Cu_7_PS_6_, κ_ph_^(1)^ = 0.05 Wm^−1^ K^−1^ at the temperature 300 K (Fig. 7b). For the calculations, the default displacement amplitude of atoms *A* = 0.03 Å was used. We have checked the convergence of the calculated coefficient κ_ph_^(1)^ in relation to the inverse lattice *q*-mesh size and the cutoff distance of the interatomic interaction. The convergence test in relation to the influence of the *q*-mesh size on the κ_ph_^(1)^ coefficient, presented in Fig. 7b, confirms the validity of the calculated κ_ph_^(1)^ coefficient for Cu_7_PS_6_. Also, when the cutoff distance *R*_cutoff_ changes from 3 Å to the nonlimited value in the unit cell box 9.6 × 9.6 × 9.6 Å^3^ the coefficient κ_ph_^(1)^ changes in the range of 8% of the mean κ_ph_^(1)^ value, but this dependence was found to be not monotonous. Using the same version of the Phono3py code (v.2.0.0) the calculated coefficient of the lattice thermal conductivity of silicon crystal has been found to be close to the reference value κ_ph_^(1)^ = 130 Wm^−1^ K^−1^ (Fig. 7b). This may be regarded as the confirmation of the correctness of the calculations using the applied Phono3py version. On the other hand, we are aware that the extremely small value of the coefficient κ_ph_^(1)^ = 0.05 Wm^−1^ K^−1^ may partly be a result of the negative acoustic frequency ν and negative derivative dν/d*k* close to the Γ-point (Fig. 7a), which may decrease the acoustic velocity in the crystal.

The electron part of the thermal conductivity κ_e_ of the extrinsic Cu_7_PS_6_ (Fig. 6a) is much smaller than the lattice one κ_ph_^(1)^ of the nominal crystal (Fig. 7b). Taking into account the relation κ_ph_^(1)^ >  > κ_e_, the values κ_ph_^(1)^ were used for the calculation of the thermoelectric figure of merit *ZT* presented in Fig. 6b. According to these estimations, the value *ZT* of the extrinsic Cu_7_PS_6_ may be of practical interest (*ZT* > 0.1) for the heavy doped *n*-type Cu_7_PS_6_ of the electrons concentration *n* ≥ 10^18^ cm^−3^ and the holes concentration *n* ≥ 10^19^ cm^−3^.

**Figure 8:**
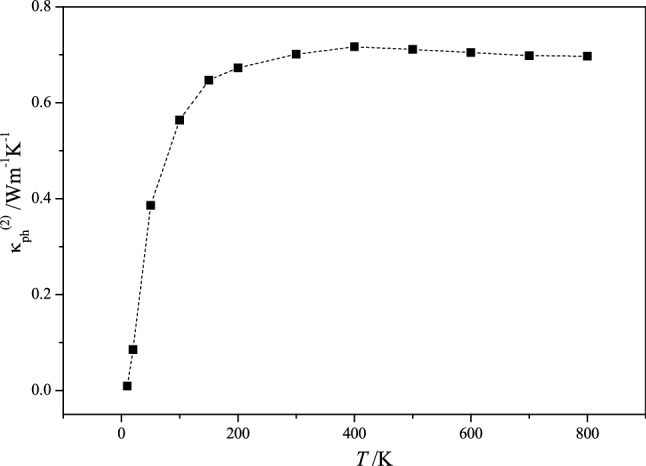
Temperature dependence of lattice thermal conductivity coefficient κ_ph_^(2)^ of Cu_7_PS_6_ obtained by using the results of molecular dynamics calculations and relation (8).

Temperature dependence of the coefficient of thermal conductivity κ_ph_^(2)^ (8) of Cu_7_PS_6_ calculated in the framework of the diffusion model^32^ on the basis of the MD calculations is presented in Fig. 8. In the temperature range of 300–800 K, the coefficient of thermal conductivity κ_ph_^(2)^ of Cu_7_PS_6_ remains almost constant, κ_ph_^(2)^ = 0.7 W/(m K). The calculated value κ_ph_^(2)^ = 0.7 W/(m K) for Cu_7_PS_6_ is of the same order of magnitude as the experimentally measured value κ_ph_^(exp)^ ≈ 0.25 – 0.33 Wm^−1^ K^−1^^41^. The coefficient of lattice thermal conductivity κ_ph_^(2)^ = 0.7 W/(m K) is one order of magnitude larger than the similar value κ_ph_^(1)^ = 0.05 W/(m K) obtained by utilizing Phono3py code (Fig. 7b). Both above mentioned calculated values of the lattice thermal conductivity κ_ph_^(1)^ = 0.05 W/(m K) and κ_ph_^(2)^ = 0.7 W/(m K) of Cu_7_PS_6_ at the temperature 300 K may in principle be acceptable because they correspond to the figure of merit *ZT* calculated for different carrier concentration and temperatures (Fig. 6b). It is hard to compare the calculated here the thermoelectric figure of merit *ZT* of the extrinsic Cu_7_PS_6_ and the corresponding experimental results^41^ because the latter ones relate to the mixed type of electric conductivity covering the activation-like and scattering-like mechanisms.

## Methods of calculations

A study of the electronic properties of Cu_7_PS_6_ crystals was performed in the framework of the density functional theory (DFT) using the VASP code^18–23^ and PAW pseudopotentials^23^. In view of the relatively large conventional unit cell of the crystal (*a* = *b* = *c* = 9.59 Å, space group no. 198), the dispersion interactions (van der Waals) have been taken into account in the form of the opt-B86b functional^24^.

Before the production calculations of the crystal studied, the total energy convergence tests were performed in relation to the cutoff energy of plane waves (*E*_cutoff_) and the *k*-points grid used. Finally, the cutoff energy *E*_cutoff_ = 390 eV and the *k*-points grids from 6 × 6 × 6 to 10 × 10 × 10 of the reciprocal lattice have been used, depending on the type of the calculation task.

The thermoelectric properties of Cu_7_PS_6_ were calculated using the recently published AMSET code^30^ developed on the basis of VASP and BoltzTraP2^16,17^. The BoltzTraP2 code is based on the semi-classical Boltzmann transport theory^42^ with the density of electron states (DOS) only as input. Therefore, before the calculations of thermoelectric properties using AMSET and BoltzTraP2 codes, the DFT calculations of the electronic band structure were performed using the VASP code. Apart from the calculation of the Seebeck coefficient α, electric conductivity per relaxation time σ/τ and electron thermal conductivity per relaxation time κ_e_/τ, performed using the BoltzTrap2, the calculation of the electron/hole scattering factor may be realized by using the AMSET code (Ab initio Scattering and Transport). In the AMSET code, different models of the electron/hole scattering are implemented (e.g. acoustic deformation potential, polar optical phonon scattering, and ionized impurity), which permits to obtain of the reliable values of the carrier relaxation time in the material studied. Due to this, the more reliable thermoelectric values necessary to compare with the corresponding experimental ones may be obtained.

The dispersion of the phonon bands of Cu_7_PS_6_ crystal was calculated using the Phonopy code^37^.

The coefficient of lattice thermal conductivity κ_ph_^(1)^ of Cu_7_PS_6_ was calculated using the VASP and Phono3py codes^33^. To obtain the reasonable value of κ_ph_ coefficient one generally should not use a small unit cell^33^. Due to the relatively large unit cell dimensions of Cu_7_PS_6_ crystal (*a* = *b* = *c* = 9.6 Å) and the number of atoms (*N*_a_ = 56) the supercell 1 × 1 × 1 has been used for the corresponding calculations. At the cubic symmetry *P*2_1_3 of Cu_7_PS_6_, the number of the VASP structure files (and corresponding runs of the SCF electron calculations), necessary for the calculation of the FORCES_FC3 file for the Phono3py calculations, is equal to 9436. This indicates that the use of larger supercells, e.g. 2 × 2 × 2, together with the dense *k*-point grid for this purpose may not be acceptable because of the huge amount of the necessary computational resources.

To obtain better insight into the phonon-associated effects in Cu_7_PS_6_ crystal the molecular dynamics calculations have been performed using the VASP code at different temperatures in the range of 20–800 K. The results of MD obtained were elaborated using the nMoldyn 3.0 code^43^.

## Conclusions

The Burstein–Moss effect is shown to be successfully utilized for the calculation of the electron effective mass dependence on the electric carrier concentration of the extrinsic type in Cu_7_PS_6_ crystal. An increase of the absolute value of the electron effective mass in Cu_7_PS_6_ with an increase of the electrons and holes concentration in the range *n* > 3 × 10^20^ cm^−3^ has been detected, which should be taken into account in the calculation of the thermoelectric properties depending on the electric carriers mobility. The polar optical phonon scattering of the electric carriers is the predominant one in Cu_7_PS_6_. The mechanism of the ionized impurity scattering prevails only for the heavy doped Cu_7_PS_6_ (*n* ≥ 10^21^ cm^−3^). The weak chemical bonding of copper atoms in Cu_7_PS_6_ leads to the extremely small coefficient of the lattice thermal conductivity κ_ph_^(1)^ ≈ 0.05 Wm^−1^ K^−1^ obtained from the calculated phonon lifetime using the Phono3py code. The relatively high temperature stimulated mobility of the copper atoms in comparison to the phosphorous and sulfur ones found in Cu_7_PS_6_ crystal justifies the applying of the diffusion-based model of thermal conductivity. In the framework of this model, the calculated coefficient of lattice thermal conductivity of Cu_7_PS_6_, κ_ph_^(1)^ ≈ 0.7 Wm^−1^ K^−1^, is close to the experimental one κ_exp_ ≈ 0.2–0.3 Wm^−1^ K^−1^. For the heavy doped Cu_7_PS_6_ of the carrier concentration about 1 × 10^19^–1 × 10^21^ cm^−3^, the calculated values of the thermoelectric figure of merit are comparable with the corresponding experimental ones obtained for the mixed type of electric conductivity covering the activation-like and scattering-like mechanisms.

## Supplementary Information

Supplementary Information.

Supplementary Figure 1.

Supplementary Table 1.
