# Supplementary material for: Electron, phonon and thermoelectric properties of Cu7PS6 crystal calculated at DFT level
Source: Sci Rep. 2021 Sep 24;11:19065. doi: 10.1038/s41598-021-98515-6 (PMC8463705; doi:10.1038/s41598-021-98515-6)
Supplement: Supplementary file 1 — Supplementary Information. [file 41598_2021_98515_MOESM1_ESM.docx]

**Electron, phonon and thermoelectric properties of Cu_7_PS_6_ crystal calculated at DFT level**

B. Andriyevsky, I. E. Barchiy, I. P. Studenyak, A. I. Kashuba, and M. Piasecki

**Supplementary Information**

**Effective electron mass *m*^*^ of Cu_7_PS_6_ calculated by the Effective Mass Calculator**

SI-Table 1. Averaged values of the effective masses *m*^*^ (in units of free electron mass *m*_e_) of Cu_7_PS_6_ calculated using Effective Mass Calculator [26] at different points of Brillouin Zone (Γ, R, M, X, X1) for top valence (v235 and v236) and bottom conduction (c237 and c238) bands. Averaging is performed over three diagonal components of the effective mass tensor *m*^*^_ii_ (*i* = 1, 2, 3), *m*^*^ = 3/(1/*m^*^*_11_ + 1/*m^*^*_22_ + 1/*m^*^*_33_)

|  | Γ | R | M | X | X1 |
| --- | --- | --- | --- | --- | --- |
| v234 | -0.40 | -0.29 | -2.90 | 0.69 | 0.68 |
| v235 | -1.07 | 0.29 | 10.5 | 272 | 146 |
| v236 | -25.5 | 0.29 | 10.5 | 0.65 | 0.65 |
| c237 | 0.59 | -0.73 | -0.17 | -0.09 | -0.09 |
| c238 | 0.24 | -0.750 | -0.17 | -0.08 | -0.08 |

For the *n*-type conductivity in Cu_7_PS_6_, the only positive effective masses of the conduction bands c237 and c238 at Γ-point of BZ, *m*^*Γ^_c237_ = 0.59, and *m*^*Γ^_c238_ = 0.24, may be essential. Similarly, the only negative effective masses of the valence bands v234, v235, and v236 at Γ-point of BZ, *m*^*Γ^_v234_ = -0.40, and *m*^*Γ^_v235_ = -1.07, and *m*^*Γ^_v236_ = -25.5 may primarily affect the *p*-type conductivity in Cu_7_PS_6_. The relatively large absolute value of the effective mass *m*^*Γ^_v236_ = -25.5 is caused partly by the complicated character of the band energy dispersion *E*(*k*), where the local minimum of the dependence *E*(*k*), taking place in close vicinity of Γ-point, is superposed on the maximum of *E*(*k*) in a more wide range of the wave vector *k* (see inset of Fig. 2a). Generally, at Γ-point of Brillouin zone, the absolute values of effective masses at the conduction bands c237 and c238 are smaller than those in the valence bands v234, v235, and v236 (SI-Table 1, Fig. 2a). Therefore, this feature predicts the larger *n*-type conductivity compared to the *p*-type one for close carrier concentrations *n*.

**Nonlinearity of copper atom vibrations in Cu_7_PS_6_**

The atomic mass *A* of the copper atom (*A*_Cu_ = 63.546) are twice heavier than the phosphorous (*A*_P_ = 30.974) or sulfur (*A*_S_ = 32.06) ones. This feature substantiates the observed relations of the vibration partial density of states (PDOS) for these atoms, according to which the median of PDOS frequency distribution for copper is more than three times smaller than the similar values for sulfur and phosphorous (SI-Fig. 1a). Taking into account the well-known influence of the mass *m* on the frequency ν of spring oscillations, ν = √(*k*/*m*) (*k* is the spring constant), the mentioned above feature means that the copper atom related spring constant *k*_Cu_ is more than four times smaller than the similar values *k*_S_ or *k*_P_ for sulfur and phosphorous ones. This result leads to the conclusion that copper atoms are bonded much weaker than the phosphorous and sulfur ones in Cu_7_PS_6_ crystal structure.

Additionally, a clear decrease of the frequency maximum of the vibration partial density of states for copper atoms with an increase in temperature has been revealed (SI-Fig. 1b). This decrease clear indicates the anharmonicity (nonlinearity) of the copper atoms' vibrations and the corresponding decrease of the spring constant *k*_Cu_.

(a) (b)

SI-Fig. 1. Vibration PDOS of (a) phosphorous, sulfur and copper atoms of Cu_7_PS_6_ calculated by using lattice dynamics and (b) copper atoms of Cu_7_PS_6_ calculated by using molecular dynamics at different temperatures

**Figure legend**

SI-Fig. 1. Vibration PDOS of (a) phosphorous, sulfur and copper atoms of Cu_7_PS_6_ calculated by using lattice dynamics and (b) copper atoms of Cu_7_PS_6_ calculated by using molecular dynamics at different temperatures
