# Supplementary material for: Electron, phonon and thermoelectric properties of Cu7PS6 crystal calculated at DFT level
Source: Sci Rep. 2021 Sep 24;11:19065. doi: 10.1038/s41598-021-98515-6 (PMC8463705; doi:10.1038/s41598-021-98515-6)
Supplement: Supplementary file 3 — Supplementary Table 1. [file 41598_2021_98515_MOESM3_ESM.docx]

SI-Table 1. Averaged values of the effective masses *m*^*^ (in units of free electron mass *m*_e_) of Cu_7_PS_6_ calculated using Effective Mass Calculator [26] at different points of Brillouin Zone (Γ, R, M, X, X1) for top valence (v235 and v236) and bottom conduction (c237 and c238) bands. Averaging is performed over three diagonal components of the effective mass tensor *m*^*^_ii_ (*i* = 1, 2, 3), *m*^*^ = 3/(1/*m^*^*_11_ + 1/*m^*^*_22_ + 1/*m^*^*_33_)

|  | Γ | R | M | X | X1 |
| --- | --- | --- | --- | --- | --- |
| v234 | -0.40 | -0.29 | -2.90 | 0.69 | 0.68 |
| v235 | -1.07 | 0.29 | 10.5 | 272 | 146 |
| v236 | -25.5 | 0.29 | 10.5 | 0.65 | 0.65 |
| c237 | 0.59 | -0.73 | -0.17 | -0.09 | -0.09 |
| c238 | 0.24 | -0.750 | -0.17 | -0.08 | -0.08 |
